# Sugar sweetened beverage consumption during pregnancy is associated with lower diet quality and greater total energy intake

**DOI:** 10.1371/journal.pone.0215686

**Published:** 2019-04-25

**Authors:** Ryan J. Gamba, Cindy W. Leung, Lucia Petito, Barbara Abrams, Barbara A. Laraia

**Affiliations:** 1 Department of Health Sciences, California State University, East Bay, Hayward, California, United States of America; 2 Department of Nutritional Sciences, University of Michigan School of Public Health, Ann Arbor, Michigan, United States of America; 3 Division of Epidemiology, Harvard T.H. Chan School of Public Health, Cambridge, Massachusetts, United States of America; 4 Division of Epidemiology, School of Public Health, University of California, Berkeley, Berkeley, California, United States of America; 5 Division of Community Health and Human Development, School of Public Health, University of California, Berkeley, Berkeley, California, United States of America; Imperial College London, UNITED KINGDOM

## Abstract

**Objective:**

Identify the socio-economic correlates of sugar sweetened beverage (SSB) consumption among pregnant women and analyze to what extent SSB consumption is associated with diet quality and total energy intake. Additionally, we aim to predict how diet quality scores and totally energy intakes would change if SSB consumption was artificially set to 0.

**Design:**

Repeated Cross Sectional Study.

**Setting:**

United States.

**Subjects:**

SSB consumption was estimated from 1–2 24-hour dietary recalls from 1,154 pregnant women who participated in the 1999–2006 National Health and Nutrition Examination Survey.

**Methods:**

Linear regression models were used to identify socioeconomic and demographic factors associated with SSB consumption and to assess the associations between SSB consumption and diet quality and total energy intake. Diet quality was measured with the Alternate Healthy Eating Index modified for Pregnancy (AHEI-P).

**Results:**

The mean SSB intake was 1.3 servings per day (sd 1.5). Having a household income ≤100% of the Federal Poverty Level, being born in the United States, and not being married or living with a partner were positively associated with SSB consumption. Every 12 oz. of SSBs consumed was associated with a 2.3 lower AHEI-P score (95% CI: 1.6, 2.9) and the consumption of 124 more calories (95% CI: 85, 163), after adjusting for age, country of birth, race/ethnicity, educational attainment, marital status, household income, survey year and day/s of the week the recall/s were collected. Our predictive models indicated that average AHEI-P would be 6.4 (5.4, 7.6) higher and average total energy intakes would be 203.5 calories (122.2, 284.8) lower if SSB intake was set to 0.

**Conclusions:**

SSB consumption is associated with poorer diet quality and higher total energy intake among pregnant women.

## Introduction

Sugar sweetened beverages (SSBs) are the single largest contributor of added sugars to the American diet, accounting for 45% of all added sugars consumed [1]. SSBs include non-diet soft drinks, energy drinks, sweetened frozen coffee drinks, fruitades, smoothies, sports drinks, sweetened fruit drinks and sweetened beverages, but not natural juices that may be high in sugar. The 2015–2020 Dietary Guidelines Advisory Committee recommended limiting added sugar intake to <10% of total energy intake, however the average American intake is close to 15% [1,2]. In non-pregnant populations, SSB consumption has been associated with poor diet quality [3, 4], weight gain [5], obesity [6, 7], cardiovascular disease and some cancers [8]. Among pregnant women, SSB consumption has been associated with an elevated odds for preterm delivery [9], greater weight-for-age at birth [10], and offspring obesity [11]. Despite the potentially harmful health effects of consuming SSBs both before and during pregnancy, the amount of SSBs being consumed and the correlates of consumption during pregnancy are unknown.

American pregnant women have diets that need improvement [12], and approximately half of American pregnant women gain weight in excess of the Institute of Medicine’s (IOM) guidelines (S1 Table)[13]. Excessive weight gain and poor diet quality during pregnancy lead to an array of adverse maternal and child health outcomes [14, 15], including preterm birth [16] and obesity in the mother [17] and child [18]. SSB consumption in pregnancy may lead to both excessive weight gain and poor diet quality by increasing total energy intakes [19] and replacing healthier foods in the diet [20]. However, the extent to which caloric intake and diet quality would change if women abstained from consuming SSBs is unknown. Using National Health and Nutrition Examination Survey (NHANES) data from 1999–2006, we aimed to (1) identify sociodemographic characteristics associated with SSB consumption; (2) analyze the extent to which SSB consumption is associated with diet quality and total energy intake; and (3) investigate how caloric intake and diet quality would change if all pregnant women did not consume SSBs.

## Methods

This cross-sectional study used 1999–2006 data from NHANES; an ongoing multistage survey administered by the National Center for Health Statistics that selects a nationally representative sample of the noninstitutionalized U.S. civilian population [21]. The data underlying this study are publicly available via the Centers for Disease Control website. Please see S1 File for details regarding the construction of the dataset analyzed in the present study. Waves 1999–2006 were selected because NHANES oversampled pregnant women during these cycles to ensure better estimates of this subpopulation [21]. We excluded four pregnant women because they had implausible daily total energy intakes (>6,000 calories), and two pregnant women because they had implausible SSB intakes (>10 12 oz. servings per day). The remaining pregnant women in NHANES waves 1999–2006 with complete dietary data were included in this study (n = 1,154). This secondary data analysis was considered exempt by the University of California Berkeley Institutional Review Board.

### Diet quality

The Alternate Healthy Eating Index modified for Pregnancy (AHEI-P) was used to measure diet quality from 1–2 24 hour dietary recalls [22]. From 1999–2002, NHANES collected one 24-hour dietary recall, and from 2003–2006, NHANES collected two 24-hour dietary recalls. Dietary recalls were conducted by interviewers, and when subsequent recalls were collected this was done via telephone approximately 3 to 10 days later. Diet quality was averaged across the 24-hour recalls for those who had two recalls collected (n = 563, 49%). The AHEI-P reflects a nutritious diet during pregnancy, and includes components for vegetables, fruit, ratio of white to red meat, fiber, ratio of polyunsaturated to saturated fatty acids, folate, calcium, and iron. The Alternative Health Eating Index has a category for alcohol which is not included in the AHEI-P as alcohol is not recommended for pregnancy. The AHEI-P also does not include the nuts and soy protein component because women may avoid nuts during pregnancy out of concern for allergen sensitization. The AHEI-P has three categories not observed in the AHEI; folate, iron, and calcium as these nutrients are important for healthy a healthy pregnancy [22]. Our modified index excluded the trans-fat component because consumption of trans-fats was not available in NHANES [21]. Each component contributes up to 10 points making the maximum score 80. Scoring for each component is as follows (amount for minimum score, amount for maximum score); Vegetables (servings day) (0, ≥ 5), fruit (servings day) (0, ≥ 4), ratio of white to red meat (0, ≥ 4), fiber grams/day (0, ≥ 25 grams), polyunsaturated to saturated fatty acids ratio (≤ 0.1, ≥ 1), calcium (0, ≥ 1,200 mg), iron (0, ≥ 27 mg), and folate (0, 600 mcg). Intermediate intakes are scored proportionately between 0 and 10 [22]. Supplementation is not considered in the calculation of the nutrient scores.

### Sugar sweetened beverage intake

We created a categorical SSB intake variable for the number of 12 oz. servings consumed per day (0, >0 & < 1, ≥1 & <2, ≥2) to describe the distribution of SSB consumption in the sample and determine how diet quality and total energy intake varied across different levels of SSB consumption. These values were chosen because they are easy to interpret, and values were well-distributed across the categories. Both energy-adjusted and unadjusted measures of SSB intake were examined in the analysis. Energy-unadjusted SSB intakes were used when we measured an association between SSB consumption and total energy intake, to avoid adjusting for the outcome of interest. Energy-adjusted SSB intakes were used when we measured an association between SSB consumption and AHEI-P. SSB intake was adjusted for total energy intake using the residual method [23].

### Covariates

Age (<20, 20–24, 25–30, >30), educational attainment (no high school diploma or General Education Diploma [GED]), high school diploma or GED, some college or Associates Degree (AA) degree, college degree)), race/ethnicity (non-Hispanic White, Mexican American, Other Latino, non-Hispanic Black, other/multicultural), household income (≤100% of the Federal Poverty Level (FPL), >100% & ≤200%, >200% & ≤300%, >300%), and survey year (1999–2000, 2001–2002, 2003–2004, 2005–2006) were considered a priori to be included as confounders in the associations between SSB consumption and diet quality and total energy intake because previous research indicated these variables were associated with SSB consumption in pregnancy and total energy intake and diet quality [12, 22, 24–27]. Household food security status (food secure, marginally food secure, food insecure), household size (1–2 people, 3–5 people, ≥6 people), marital status (married or living with a partner, divorced/separated/single/widowed), country of birth (born in the U.S., born outside of the U.S.), Previous number of live births (0, ≥1), and trimester (1^st^, 2^nd^, 3^rd^) were considered potential confounders. The day/s of the week the recall/s were collected (1 or 2 weekdays, 1 weekend day and 1 weekday, and 1 or 2 weekend days, where a weekend day refers to Friday, Saturday or Sunday) was included in all models to reduce the variability in SSB consumption, diet quality, and total energy intake. Household food security status was derived from the 18 question United States Department of Agriculture’s Standard Food Security Survey Module [28].

### Statistical methods

Total energy intake was measured in calories and was square-root transformed to improve normality. All measures of total energy intake reported in the manuscript were transformed back to represent calories. To ensure the sample was nationally representative, dietary sampling weights were recalculated to reflect the probability of being sampled in the 8 year period [21].

Random effect models were implemented to identify the proportion of the total variance due to between-person variability for the SSB intakes. For the descriptive statistics, the standard deviations reflect only the between-person variability in the estimates; within-person variation due to having multiple recalls has been partitioned out.

For descriptive analysis, F tests were conducted to assess the associations between SSB consumption and each variable. Wald tests were conducted on linear regression coefficients when categorical variables were assessed. To determine which of the socio-economic correlates were significantly associated with SSB consumption when all variables were considered, we conducted a linear regression that included age, country of birth, race/ethnicity, educational attainment, marital status, household income, survey year and day/s of the week the recall/s were collected. Additionally, a sensitivity analysis was conducted that included this set of variables and those not associated with SSB intake in bivariate analysis; household size, number of live births a mother had had previously, trimester, and household food security status. Similarly, linear regressions were conducted to assess the relationship between SSB consumption and AHEI-P and total energy intake and adjusted for this set of confounders.

The models implemented to analyze the extent to which SSB consumption was associated with diet quality and total energy intake were also applied to generate the population average diet quality and total energy intake values that would result from removing SSBs from the population’s diet. After analyzing the associations between SSB consumption and diet quality and SSB consumption and total energy intake in separate linear regression models, these same models were applied to generate predicted diet quality scores and total energy intakes for each individual based on their covariate profiles, and assuming they did not consume SSBs. The diet quality and energy intakes that were generated by these models under the assumption that no SSBs were consumed were then compared to the observed population average intakes.

STATA (version 12.0, StataCorp, College Station, TX) was used for all statistical analysis. This study was considered exempt from review by the University of California Berkeley’s Institutional Review Board.

## Results

**Table 1** describes the study sample and the relationships between unadjusted SSB consumption and the covariates. On average, women were 27 ± 6 years of age. Fifty-three percent of the sample was non-Hispanic White, 17% were Mexican American, 17% were non-Hispanic Black, 5% were Other Latino and 8% were of another race or multicultural. Seventy-eight percent of the women were born in the U.S. and 72% were married or living with a partner. Pregnant women in the United States consumed an average of 1.3 ± 1.5 12 ounce unadjusted servings of SSBs per day, or 15.6 oz. SSB consumption declined from 1999–2006 from 1.4 (95% CI: 1.1–1.8) to 1.1 (0.8–1.4) servings per day (Table 1). A sensitivity analysis analyzing adjusted measures of SSB intake produced consistent results.

**Table 1:** Average unadjusted sugar sweetened beverage intake of pregnant women by sociodemographic & descriptive characteristics in the National Health and Nutrition Examination Survey from 1999–2006 (n = 1,154).

Covariates^1^	n (%)	12 oz. Servings of Sugar Sweetened Beverages^2^^,^^3^
Whole Population	1154 (100)	1.3 ± 0.9
Age (years)*		
>30	294 (27.7)	1.0 ± 0.8
25–30	327 (30.6)	1.4 ± 1.0
20–24	331 (30.0)	1.4 ± 0.9^a^
<20	202 (11.8)	1.8 ± 1.2^c^
Household Size		
1 or 2 persons	266 (24.8)	1.3 ± 0.9
3–5 persons	696 (62.9)	1.2 ± 0.9
≥6 persons	192 (12.3)	1.7 ± 1.2
Country of birth*		
Born in the U.S.	839 (77.6)	1.5 ± 1.0
Not born in the U.S.	315 (22.4)	0.7 ± 0.7^c^
Race/Ethnicity*		
Non-Hispanic White	515 (53.3)	1.3 ± 0.9
Mexican American	337 (16.7)	1.1 ± 1.0
Other Latino	56 (4.9)	1.0 ± 0.8
Non-Hispanic Black	178 (17.3)	1.7 ± 1.0^a^
Other/Multiracial	68 (7.9)	1.3 ± 0.8
Educational Attainment*		
College degree	260 (27.6)	1.1 ± 0.8
Some college or AA degree	285 (30.4)	1.4 ± 0.9
High school diploma/GED	246 (19.5)	1.4 ± 1.0
No High School diploma/GED	362 (22.5)	1.5 ± 1.1^a^
Missing	1 (<0.1)	-
Marital Status*		
Married/living with a partner	828 (72.1)	1.1 ± 0.8
Divorced/separated/single/widowed	284 (23.3)	1.9 ± 1.1^c^
Missing	42 (4.6)	-
Household Income^4^*		
>300%	391 (35.7)	1.1 ± 0.9
200% ≤ 300%	154 (17.5)	1.4 ± 0.8
100% ≤ 200%	246 (20.1)	1.4 ± 1.0
≤100%	285 (19.8)	1.7 ± 1.1^b^
Missing	78 (7.0)	-
Household Food Security Status		
Food secure	804 (75.4)	1.3 ± 0.9
Marginally food secure	121 (10.4)	1.3 ± 0.9
Food insecure	179 (10.7)	1.5 ± 1.2
Missing	50 (3.5)	-
Number of Live Births		
0	57 (6.0)	1.3 ± 0.8
≥1	1,097 (94)	1.3 ± 0.9
Trimester		
1st Trimester	194 (19.7)	1.3 ± 0.8
2nd Trimester	405 (31.6)	1.2 ± 1.0
3rd Trimester	386 (29.7)	1.4 ± 1.0
Missing	169 (19.0)	-
Year the Survey was collected		
1999–2000	269 (24.5)	1.4 ± 1.1
2001–2002	322 (24.5)	1.3 ± 1.0
2003–2004	245 (23.0)	1.5 ± 0.9
2005–2006	318 (28.0)	1.1 ± 0.8
Recall collection day/s		
1 or 2 weekdays	338 (44.6)	1.3 ± 0.8
1 weekday & 1 weekend day	417 (30.2)	1.3 ± 0.9
2 weekend days	399 (25.2)	1.4 ± 1.1

^1^ Covariates labeled with an * indicate that the variable was significantly associated with unadjusted SSB consumption p<0.20 according to an f-test of independence

^2^ Values represent the average number of 12 oz. servings ± the standard deviation

^3^ Wald tests on linear regression coefficients were implemented to determine if the observed value was significantly different than the reference value; the first row below each covariate name represents the reference value

^4^ Measured as a percent of the Federal Poverty Level

^a^ Significantly different than the reference value at p<0.05

^b^ Significantly different than the reference value at p<0.01

^c^ Significantly different than the reference value at p<0.001

The average AHEI-P score was 44.5 (43.3–45.7) and the average total energy intake was 2,272 calories (2,176–2,369). **Table 2** highlights the negative association between SSB consumption and diet quality and the positive association between SSB consumption and total energy intake. Women who did not consume SSBs had an average AHEI-P of 52.6 (49.5, 55.7) and an average total energy intake of 2,072 calories (1,925–2,219). In contrast, those who consumed 2 or more 12 oz. servings of SSBs had a lower average AHEI-P score of 39.7 (37.6–41.8), and a higher average total energy intake of 2,492 calories (2,354–2,630) (p<0.001).

**Table 2:** Average Alternative Healthy Eating Index modified for Pregnancy (AHEI-P) score and total energy intake by level of sugar sweetened beverage (SSB) intake for pregnant women in the National Health and Nutrition Examination Survey 1999–2006 (n = 1,154).

12 oz. Servings of SSBs	n^1^	Average Observed AHEI-P^2^ (95% CI)	n^3^	Average Observed total energy intake (95% CI)
0	131	52.6 (49.5, 55.7)	307	2072 (1925, 2219)
0 - <1	421	46.8 (45.0, 48.6)^a^	255	2181 (1900, 2461)
1 - <2	303	41.7 (40.0, 43.4)^b^	287	2364 (2257, 2470)^a^
≥2	299	39.7 (37.6, 41.8)^b^	305	2492 (2354, 2630)^b^

^1^ This n represents the number of individuals who were in each category of SSB consumption when SSB values adjusted for energy were assessed

^2^ Wald tests on linear regression coefficients were implemented to determine if a value was significantly different than the AHEI-P score or total energy intake among those who did not consume SSBs

^3^ This n represents the number of individuals who were in each category of SSB consumption when SSB values were not adjusted for energy were assessed

^a^ Significantly different than the value for those who did not consume SSBs at p<0.01

^b^ Significantly different than the value for those who did not consume SSBs at p<0.001

Our first aim was to identify the socio-economic correlates of SSB consumption. Our linear regression analysis found having an income >300% of the federal poverty level compared to having an income ≤100% of the FPL was negatively associated with SSB consumption (p ≤ 0.05), as was being born outside of the U.S. compared to being born in the U.S. and being married or living with a partner compared to being single, widowed, divorced, or separated (**Table 3**). Marital status was no longer a significant predictor of SSB consumption in our sensitivity analysis, when variables not associated with SSB consumption in bivariate analysis were included in the model; household size, number of live births a mother had previously, trimester, and household food security status.

**Table 3:** Associations between sociodemographic and descriptive factors and unadjusted sugar sweetened beverage (SSB) intake among pregnant women in the National Health and Nutrition Examination Survey 1999–2006 (n = 1,154).

Characteristics	12 oz. Servings of SSBs^1^^,^ ^2^
Age (years)	
>30	reference
25–30	0.2 (-0.1, 0.5)
20–24	0.1 (-0.3, 0.4)
<20	0.1 (-0.4, 0.6)
Country of birth	
Born in the U.S.	reference
Not born in the U.S.	-0.7 (-1.0, -0.4)^b^
Race/Ethnicity	
Non-Hispanic White	reference
Mexican American	-0.1 (-0.4, 0.3)
Other Latino	-0.1 (-0.6, 0.4)
Non-Hispanic Black	-0.0 (-0.5, 0.4)
Other/Multiracial	0.2 (-0.6, 1.0)
Educational attainment	
College degree	reference
Some college or AA degree	0.0 (-0.4, 0.5)
High school diploma/GED	0.1 (-0.4, 0.5)
No high school diploma/GED	0.1 (-0.4, 0.5)
Marital Status	
Married/living with a partner	reference
Divorced/separated/single/widowed	0.6 (0.1, 1.0)^a^
Household Income^3^	
>300%	reference
200% ≤ 300%	0.3 (-0.1, 0.8)
100% ≤ 200%	0.3 (-0.2, 0.8)
≤100%	0.5 (0.0, 0.9)^a^
Year the Survey was collected	
1999–2000	reference
2001–2002	-0.1 (-0.5, 0.3)
2003–2004	0.1 (-0.4, 0.5)
2005–2006	-0.3 (-0.8, 0.2)

^1^ Values represent the ß coefficient and 95% confidence interval from the regression model where SSB was the outcome and age, country of birth, race/ethnicity, educational attainment, marital status, household income, and day of the week the recall/s were collected were all included as independent variables in the same model

^2^ Wald tests on linear regression coefficients were implemented to determine if the observed value was significantly different than the reference value

^3^ Measured as a percent of the Federal Poverty Level

^a^ Significant at p<0.05

^b^ Significant at p<0.001

Our second aim was to assess the relationship between SSB’s and AHEI-P and total energy intake. Consuming an additional 12 oz. of SSBs was associated with a 2.3 point lower AHEI-P score (1.6, 2.9), adjusting for potential confounders (**Table 4**). The linear regression model analyzing the association between SSB consumption and total energy intake showed that consuming an additional 12 oz. of SSBs was associated with consuming an additional 124 calories per day (85–163), adjusting for potential confounders (**Table 4**).

**Table 4:** Sugar lweetened beverage consumption and associated changes in energy consumption and Alternative Healthy Eating Index modified for Pregnancy (AHEI-P) scores among pregnant women in the National Health and Nutrition Examination Survey 1999–2006 (n = 1,154).

Change in SSB Consumption^1^	Changes in AHEI-P^2^	Changes in energy intake (calories)^3^
Consuming 1 additional 12 oz of SSBs	-2.3 (-1.6, -2.9)^a^	124 (85, 163)^a^
Changing SSB consumption to 0 for the whole population	6.4 (5.4, 7.6)^a^	-203.5 (-122.2, -284.8)^a^

^1^ Linear regression model adjusted for age, country of birth, race/ethnicity, educational attainment, marital status, household income, survey year and day/s of the week the recall/s were collected

^2^ Linear regression models assessed energy adjusted SSB intake

^3^ Linear regression models assessed energy unadjusted SSB intake

^a^ Significantly different than 0 at p<0.01

Finally, our last aim was to investigate how caloric intake and diet quality would change if all pregnant women did not consume SSBs. Compared to the observed average values of diet quality and total energy intake, when we set the model to reflect no SSB consumption, the average diet quality score was 6.4 points higher (5.4–7.6) and the average total energy intake was 203.5 calories lower (122.2, 284.8) (**Table 4**).

## Discussion

Pregnant women were found to consume more SSBs on average than women who were not pregnant. On average, pregnant women consumed 15.6 oz. of SSBs per day, which equates to approximately 176 calories. A report analyzing survey data from NHANES 2005–2006 found women aged 20–39 consumed an average of 138 calories, or roughly 13 oz. of sugar drinks (defined similarly to SSBs) per day, which is about 2.6 oz. less than observed in this sample [29]. Although pregnant women typically need to consume additional calories during pregnancy, the observed increase in SSB consumption during pregnancy is worrying considering the adverse health effects associated with SSB consumption [8–10, 30, 31] and the importance of diet during pregnancy [14, 16, 22, 32].

Income, country of birth and marital status were found to be predictive of SSB consumption. Our finding that having a lower income predicts higher SSB consumption is consistent with past research [24, 25, 33], however studies have not previously identified country of birth and marital status as correlates of SSB consumption. This is likely due to these factors not being considered as potential correlates in previous studies [24, 25, 33]. Of the 2 studies that analyzed the association between country of birth and SSB consumption, neither found significant associations with SSB intake [34, 35]. The discrepancy in findings may because these studies analyzed populations from Mexico, which has very high per capita SSB intake [36]. Additionally, they did not study pregnant populations, and women born outside of the United States may have culturally specific diets they adhere to during pregnancy that do not typically include SSBs [37]. Marital status may influence SSB consumption through stress and loneliness, as both have been positively associated with SSB consumption [38–40]. In future studies, country of birth and marital status should be considered as independent correlates of SSB consumption during pregnancy.

Consuming an additional 12 oz. SSB was associated with consuming an additional 124 (85–163) calories per day, which is consistent with past research showing consumption of SSBs may lead to consuming extra calories [6, 7]. Similar results were found among 3,098 children and adolescents from NHANES, where the consumption of one 12 oz. SSB was associated with consuming an additional 159 calories per day [41]. Research analyzing how SSBs may engender weight gain suggests caloric compensation is greater when calories are ingested as food compared to liquids; when liquid calories are consumed along with calories from food, people are likely to consume more calories than if all the calories were consumed via foods [42–44]. Additionally, compelling evidence indicates substituting water for SSBs leads to lower total energy intakes and weight loss [45–47], which further supports the research on differential energy adjustment. Given that liquid calories may be treated differently by the body than calories from solid foods during pregnancy [42–44] a reduction in SSB consumption and the associated lower total energy intake may help lead to appropriate total energy intake and gestational weight gain.

We observed an average AHEI-P score of 44.5 (43.3, 45.7), which is indicative of a diet that needs improvement. Consuming an additional 12 oz. serving of SSBs was associated with a 2.3 point lower AHEI-P score (1.6, 2.9), which is disconcerting given past research has shown a 5 point improvement in AHEI-P is associated with lower blood glucose levels and lower odds of having preeclampsia during pregnancy [22]. There is no component for SSB intake in the AHEI-P, which means the lower diet quality score associated with consuming more SSBs reflects lower scores in the other dietary components. This is in part because those who are likely to consume SSBs are also more likely to consume other unhealthy food choices that are associated with lower diet quality [48, 49]. The negative association between SSB consumption and diet quality may also be because the calories ingested from SSBs are preventing the consumption of healthier foods. Although previously mentioned studies [31–33] indicate there is a larger adjustment by the body in response to calories ingested as foods compared to liquids, there is still a weak caloric compensation for calories ingested as liquids, [50] especially when consumed without food [51]. Therefore, if women no longer consumed SSBs, the subsequent caloric deficit could be filled in part by healthier foods.

To further understand the extent to which SSB consumption is associated with poor diet quality and higher total energy intake, we estimated the difference in diet quality and total energy intake by creating a prediction model using our regression coefficients for high and low levels of SSB intake. If pregnant women who currently consume SSBs no longer consumed any SSBs, the average AHEI-P score of the population would be substantially improved and the caloric intake would be lower. Although the changes in diet quality and total energy intake are significant, these models assume that the changes in SSB intake occur while no other dietary or lifestyle changes are made, which is an unverifiable assumption. Additionally, it is difficult to contextualize the lower total energy intake associated with not consuming SSBs because we do not have information on the women’s pre-pregnancy body mass indices, and therefore do not know which women are on pace to gain excessive weight. However, if we assume that women who are gaining excessive weight consume SSBs and that lower total energy intakes lead to smaller gestational weight gain, then our results suggest a decrease in SSB consumption may lead to a reduced prevalence of excessive gestational weight gain. Observing a significant negative association between SSB consumption and diet quality, and a significant positive association between SSB consumption and total energy intake supports the hypothesis that reducing SSB consumption during pregnancy may help lead to appropriate weight gain and better health outcomes.

NHANES is a cross-sectional study, which prevents determining causality. Social desirability bias may have influenced women to report lower intakes of SSB consumption, especially among women with higher BMI’s [52]. This may have biased our results to underestimate the true difference in diet quality and energy intakes among SSB consumers and non-consumers. Estimating usual energy intake from one or two dietary recalls can be problematic given the day to day variability in food consumption, an individual’s likelihood of changing their intake in anticipation of the recall, and its dependence on the memory, cooperation, and communication ability of the subject [53]. Despite these limitations, the extensive data available in NHANES allowed us to analyze an array of variables which may have limited unmeasured confounding. Additionally, this is one of the first studies to analyze how SSB consumption is associated with diet quality and total energy intake among pregnant women.

During pregnancy, income, country of birth and marital status are associated with SSB intake. A greater intake of SSBs during pregnancy was associated with poorer diet quality and greater total energy intakes. When setting the population SSB consumption to 0, we observed the average AHEI-P to be significantly higher and the average total energy intake to be significantly lower. Future research should continue to assess the correlates of SSB consumption during pregnancy so we can better identify high consumer populations to tailor public health interventions.

## Supporting information

S1 Table Institute of Medicine Weight Gain Recommendations for Pregnancy. (DOCX)

S1 File Data Availability Information. (DOCX)
