# Supplementary material for: Sugar sweetened beverage consumption during pregnancy is associated with lower diet quality and greater total energy intake
Source: PLoS One. 2019 Apr 25;14(4):e0215686. doi: 10.1371/journal.pone.0215686 (PMC6483237; doi:10.1371/journal.pone.0215686)
Supplement: S1 Table — (DOCX) [file pone.0215686.s001.docx]

**Table S1.** Institute of Medicine Weight Gain Recommendations for Pregnancy^1^

| **Prepregnancy Weight  Category** | **Body Mass Index*** | **Recommended  Range of  Total Weight (lb)** ^**^ |
| --- | --- | --- |
| Underweight | Less than 18.5 | 28–40 |
| Normal Weight | 18.5–24.9 | 25–35 |
| Overweight | 25–29.9 | 15–25 |
| Obese (includes all classes) | 30 and greater | 11–20 |

^1^American College og Obstetricians and Gynecologists. Weight gain during pregnancy. Committee opinion no. 548. Obstetrics and gynecology. 2013;121(1):210-2.

*Body mass index is calculated as weight in kilograms divided by height in meters squared or as weight in pounds multiplied by 703 divided by height in inches.
^**^Calculations assume a 1.1–4.4 lb. weight gain in the first trimester.
Modified from Institute of Medicine (US). Weight gain during pregnancy: reexamining the guidelines. Washington, DC. National Academies Press; 2009. 2009 National Academy of Sciences.
