# Supplementary material for: Sugar sweetened beverage consumption during pregnancy is associated with lower diet quality and greater total energy intake
Source: PLoS One. 2019 Apr 25;14(4):e0215686. doi: 10.1371/journal.pone.0215686 (PMC6483237; doi:10.1371/journal.pone.0215686)
Supplement: S1 File — (DOCX) [file pone.0215686.s002.docx]

Supporting Information File: Data Availability Information

To reconstruct the dataset you need to do the following: To access the data, you will need to download demographics data, dietary data, and questionnaire data and merge them for the years 1999-2006 from the Centers for Disease Control Website: <https://wwwn.cdc.gov/nchs/nhanes/>

1999-2000, 2001-2002, 2003-2004, 2005-2006 Demographic Variables & Sample Weights 1999-2000, 2001-2002, 2003-2004, 2005-2006 Dietary Data – Individual Foods (First & Second Day when applicable) 1999-2000, 2001-2002, 2003-2004, 2005-2006 Dietary Data – Total Nutrient Intakes (First & Second Day when applicable) 1999-2000, 2001-2002, 2003-2004, 2005-2006 Dietary Data –Dietary Interview Technical Support File - Food Codes Format File 2003-2004, 2005-2006 Dietary Data - Dietary Interview Technical Support File - Modification Codes 1999-2000, 2001-2002, 2003-2004, 2005-2006 Questionnaire Data – Food Security 1999-2000, 2001-2002, 2003-2004, 2005-2006 Questionnaire Data – Reproductive Health

The following variables are needed to reconstruct the dataset.

Demographics
SEQN - Respondent sequence number
SDDSRVYR - Data Release Number
RIDSTATR - Interview/Examination Status
RIDAGEYR - Age at Screening Adjudicated - Recode
RIDRETH1 - Race/Ethnicity - Recode
DMDBORN - Country of Birth - Recode
DMDYRSUS - Length of time in US
DMDEDUC3 - Education Level - Children/Youth 6-19
DMDEDUC2 - Education Level - Adults 20+
DMDEDUC - Education - Recode (old version) DMDMARTL - Marital Status DMDHHSIZ - Total number of people in the Household INDFMPIR - Family PIR RIDEXPRG - Pregnancy Status at Exam - Recode WTINT2YR - Full Sample 2 Year Interview Weight WTMEC2YR - Full Sample 2 Year MEC Exam Weight SDMVPSU - Masked Variance Pseudo-PSU SDMVSTRA - Masked Variance Pseudo-Stratum

Dietary Data – Individual Foods

SEQN - Respondent sequence number
DR1ILINE - Food/individual component number
WTDRD1 - Dietary day one sample weight
WTDR2D - Dietary two-day sample weight
DR1DRSTZ - Dietary recall status
DRDINT - Number of days of intake
DR1DAY - Intake day of week
DR1CCMNM - Combination food number
DR1CCMTX - Combination food type
DR1IFDCD - USDA food code

Dietary Data – Total Nutrient Intakes

SEQN - Respondent sequence number
WTDRD1 - Dietary day one 2-Year sample weight

WTDR4YR - Dietary day one 4-Year sample weight

DRDDRSTS - Dietary recall status

DRDAINMD - Assigned mode of interview

DRDINTMD - Mode of interview DRDDAY - Intake day of the week DRXTKCAL - Energy (kcal)

DRXTTFAT - Total fat (gm)

DRXTSFAT - Total saturated fatty acids (gm)

DRXTMFAT - Total monounsaturated fatty acids (gm)

DRXTPFAT - Total polyunsaturated fatty acids (gm)

DRXTFOLA - Total Folate (mcg) DRXTCALC - Calcium (mg)

DRXTIRON - Iron (mg)

DRXTALCO - Alcohol (gm)

Food Security
SEQN - Respondent sequence number
HHfdsec - Household food security category

Reproductive Health
SEQN - Respondent sequence number
RHQ140 - Think that you are pregnant now?
RHQ150 - What month of pregnancy are you in?
RHD170 - # pregnancies resulting in live births
